# Correlations between the viral loads and symptoms in the SARS‐CoV‐2‐infected patients

**DOI:** 10.1002/mco2.324

**Published:** 2023-07-04

**Authors:** Xiaomin Wang, Shaolin Ma, Bing Zhao, Ganxiu Deng, Huiyuan She, Kailiang Xu, Lipeng Hao, Yiming Deng, Qiang Li, Zuoren Yu, Xiaoping Zhu

**Affiliations:** ^1^ Department of Respiration Shanghai East Hospital Tongji University School of Medicine Shanghai China; ^2^ Microbiology testing laboratory Shanghai Pudong Center for Disease Control and Prevention Shanghai China; ^3^ Department of Infectious Diseases Shanghai Pudong Gongli Hospital Shanghai China; ^4^ Department of Respiration Shanghai Seventh People's Hospital Shanghai China

Dear Editor,

COVID‐19 pandemic has brought an estimated 676 million cases and 6.88 million deaths until March 2023. Multiple waves of outbreaks have been caused by SARS‐CoV‐2 and its mutants, such as alpha, beta, gamma, delta, and omicron. It remains a big challenge to limit the rapid spread mainly due to our poor understanding of the pathological mechanisms.

Upon infection, SARS‐CoV‐2 replicates in the host cells, which does not have a standard rate or pattern. As such, the incubation period and viral load may vary between patients. Amplification of the virus fragment has been recommended by World Health Organization to determine the viral load in COVID‐19 patients,^1^ in which the cycle threshold (Ct) value is inversely related to the gene expression level or the viral load in the tested samples. The faster the virus replicates, the smaller the Ct value will be. SARS‐CoV‐2 viral load can serve as a marker of infection progress.^2^ High level of viral load was usually detected at the upper respiratory tract in the first week after diagnosis of patient.^2^, ^3^ An analysis of 323 samples from 76 COVID‐19 patients indicated higher level of viral load in sputum in the early and progressive stages than that in the recovery stage (46,800 ± 17,272 vs. 1252 ± 1027 copies/test, *p* < 0.001). The comorbidity rate was significantly higher in symptomatic patients (29.1%) than that in asymptomatic patients (9.3%).^4^ The viral load lasted longer in severe COVID‐19 patients than those with mild symptom.^3^ In the current study, different types of specimens from COVID‐19 patients were collected for the viral load examination, followed by the correlation analysis between the viral loads and ages, sample types, disease stage, lung infection, and immune response.

As shown in Table S1, information including age, symptoms, laboratory examination, and computed tomography images was indicated in 77 patients diagnosed with COVID‐19 in Shanghai according to the seventh edition of the Chinese Clinical Guidance for COVID‐19 Pneumonia Diagnosis and Treatment in early 2020, when there was no vaccine against SARS‐CoV‐2 available. Since all of the severe or critical patients were assigned to hospitalize at a clinical center equipped with ventilators where we were not able to collect the patients’ samples, all the 77 cases we recruited were classified to mild disease (i.e., nonpneumonia or mild pneumonia and Computed Tomography (CT) scores less than 12), in which 85.7% had a fever at the time of admission (median 38.5°C, IQR 38.0−39.0°C), 49.4% had cough, 36.4% had fatigue or muscle weakness, 9.1% had diarrhea, and 5.2% of the patients did not show imaging abnormality of X‐ray in the lung. The average number of days from symptom onset to diagnosis was 4.72 (range 2−6). We defined those patients at day 1 to day 4 after symptom onset as early stage, days 5 to day 8 as middle stage, and days 9 and after as late stage.

First, we analyzed the dynamic change of the viral loads in the SARS‐CoV‐2‐infected patients. Figure 1A illustrated the overall profile of Ct values of the patients from day 1 to day 9 after symptom onset. The lowest Ct values were observed in the patients at the early stage, indicating the highest viral loads, in which the geometric mean Ct was 28.75 with 95% confidence interval (CI) 19.25−32.30. It was 35.45 with 95% CI 32.63−37.73 for the patients at the middle stage, and 34.47with 95% CI 27.45−39.39 for the patients at the late stage. There was no significant difference of Ct values between the patients at middle stage and late stage. These data demonstrated the highest viral loads at the early stage of the disease in patients with COVID‐19.

**FIGURE 1:**
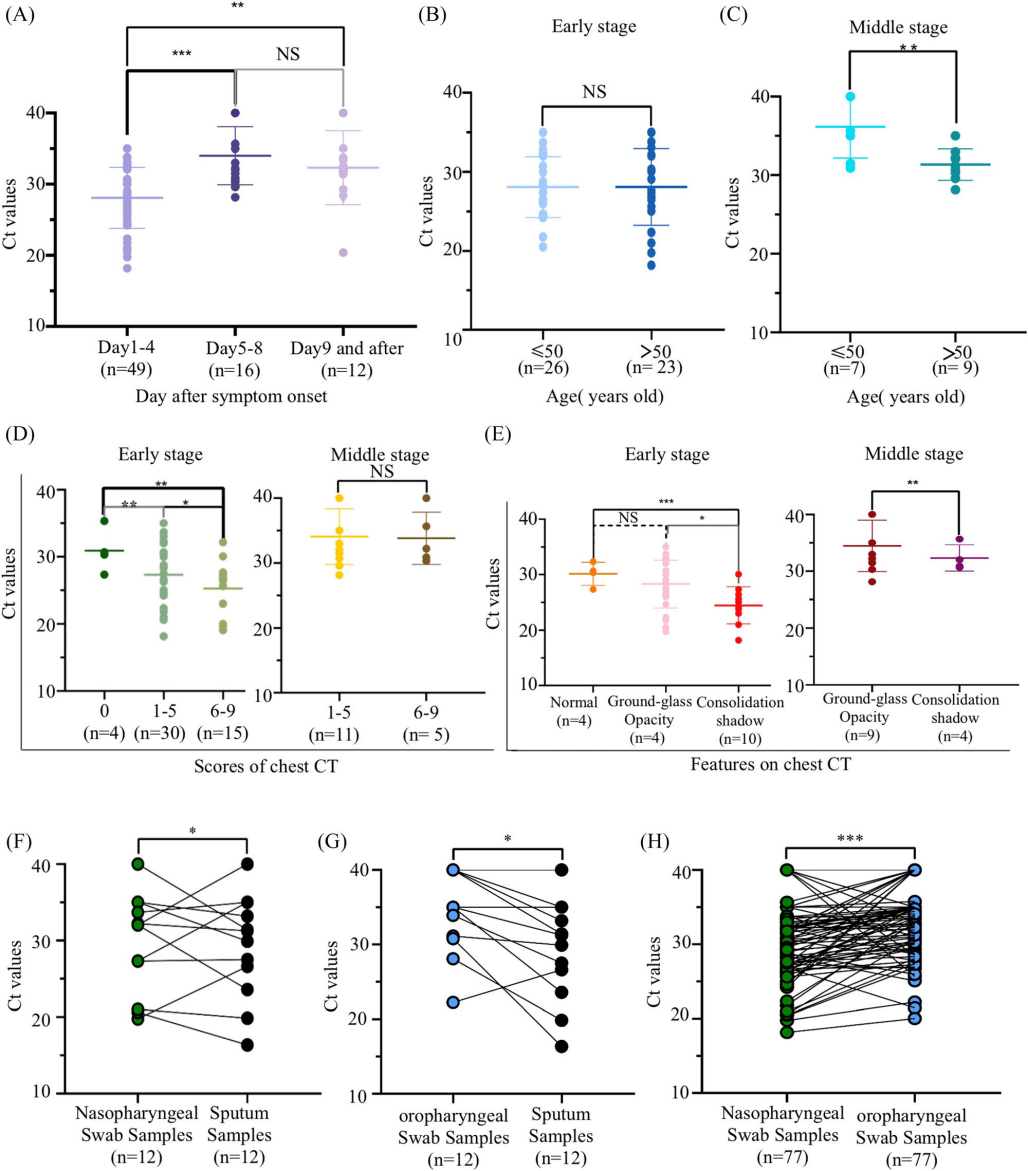
Correlations between the viral loads and symptoms. (A) Dynamic changes of the viral load (Ct Values) at different stages of the SARS‐CoV‐2‐infected patients. (B and C) Comparison of the Ct Values between the patients less than 50 years old and those over 50 years old at the early stage (B) and middle stage (C). (D) Correlation analysis of the Ct values with the computed tomography (CT) scores in the lung of patients at the early stage and the middle stage. (E) Ct values in the patients with consolidation shadow or ground glass opacity images in the lung at the early stage and the middle stage. (F and G) Comparison of the Ct Values between sputum samples (*n* = 12) and nasopharyngeal swabs (*n* = 12, F) or throat swabs (*n* = 12, G) collected from the COVID‐19 patients. (H) Comparison of the Ct values in throat swabs and nasopharyngeal swabs (*n* = 77). Ct, cycle threshold. **p ≤ 0.01, ***p ≤ 0.001. NS, nonsignificant.

Then, we analyzed correlations between the viral loads and ages, lung infection, and immune response of the patients. In view of the aged people as susceptible population to SARS‐CoV‐2, we evaluated the viral load difference between those patients younger than 50 years old and those over 50 years of age. As shown in Figure 1B, there was no significant difference of Ct values between the two groups at the early stage, while higher viral load was observed in the older patients at the middle stage of the disease (Figure 1C). In addition, higher viral loads were significantly correlated with higher CT scores in the lung (between 6−9) in those patients at the early stage (Figure 1D) and/or showing consolidation shadow CT images in the lung (Figure 1E). Consolidation shadow in the lung was defined when alveolar air was replaced by pathological fluids, cells, or tissues, manifested by an increase in pulmonary parenchymal density that obscures the margins of underlying vessels and airway walls. We also examined the concentrations of immune cells including lymphocytes and white blood cells in the blood of all the patients (Table S1 and Figures S2a–S2d), which did not show correlations with viral loads in patients at both early stage or middle stage. Taken together, our data suggested the importance of viral loads in causing severe lung infection with consolidation shadow. We demonstrated that SARS‐CoV‐2 lasted longer in the aged patients, compared with younger patients.

Last, we analyzed correlations between the viral loads and the specimen types in the patients. Both throat swab (TS) samples and nasopharyngeal swab (NS) samples were collected from all the 77 patients, followed by RT‐PCR analysis. As shown in Figures 1F–H, higher viral loads were observed in NSs than that in TSs from same patients. Additionally, we collected sputum samples from 12 patients, in which the higher viral loads were observed in sputum samples than that in either NS or TS. Furthermore, comparisons of the positive detection rate between NS and TS samples in the 77 patients were performed. As a result, 66 patients (85.7 %) were tested positive using NS samples, while 51 patients (66.2%) for TS samples (Table S2).

In conclusion, the current study found that: (1) patients with SARS‐CoV‐2 infection were highly contagious within the first 4 days after onset with the high viral loads in this timeframe; (2) although the viral loads in patients at the early stage were age independent, they decreased sooner in the patients under 50 years old than that in the patients over 50 years of age; (3) high CT scores were correlated with consolidation shadow images and high viral loads in the lungs of patients; (4) sputum samples had higher viral loads with a higher sensitivity to the RT‐PCR test method than TSs and NSs. The current study demonstrated correlations between the viral loads and the disease progression and lung infection/damage in COVID‐19 patients at different ages. It tells us when patients are highly contagious and why SARS‐CoV‐2 causes severer symptoms in old people. Moreover, this study revealed the dynamic change of the viral loads and the disease progression in those COVID‐19 patients without receiving any SARS‐CoV‐2 vaccine.

## AUTHOR CONTRIBUTIONS

X. Z. and X. W. were responsible for the content of the manuscript, including data collection and analysis. S. M., B. Z., G. D., and X. Z. conceived and designed the study. H. S., K. X., L. H., Q. L., and Y. D. coordinated to collect the samples and data. X. Z., B. Z., and X. W. analyzed and interpreted the data. X. W. and Z. Y. wrote the manuscript. All authors read and approved the final manuscript.

## CONFLICT OF INTEREST STATEMENT

The authors declared no competing interest exists.

## FUNDING INFORMATION

Not applicable.

## ETHICS STATEMENT

All the procedures were approved by the Institutional Review Board (IRB) of Shanghai East Hospital (Ethical Approval # [2023]Yanshen(030)). Written informed consents were obtained from all participants.

## Supporting information

Supporting Information

## Data Availability

The data included in this study are available upon request from the corresponding author.
