# Supplementary material for: Correlations between the viral loads and symptoms in the SARS‐CoV‐2‐infected patients
Source: MedComm (2020). 2023 Jul 4;4(4):e324. doi: 10.1002/mco2.324 (PMC10318488; doi:10.1002/mco2.324)
Supplement: Supplementary file 1 — Supporting Information [file MCO2-4-e324-s001.pdf]

Supplemental Materials and Methods

Flow Chart. A flow chart for the work design was shown in Supplemental Figure S1.

Patient Cohorts. Patients were diagnosed as COVID-19 from January 10, 2020 to March 10, 2020 in Pudong New Area, Shanghai according to the seventh edition of the Chinese Clinical Guidance for COVID-19 Pneumonia Diagnosis and Treatment. All of the patients had not received any vaccination against SARS-CoV-2. They were hospitalized in the Shanghai Public Health Clinical Center receiving regular medical treatment. Samples of throat swab, nasal swab or sputum were collected from the patients, and applied for RT-PCR analysis to determine the viral load of SARS-CoV-2. Each collected sample was put into a test tube containing 2mL of virus transport medium. The clinical information of the 77 patients were collected including age, symptoms, laboratory examination and computed tomography (CT) images. All the procedures were approved by the Institutional Review Board (IRB) of Shanghai East Hospital (Ethical Approval # [2023]Yanshen(030)).

Lung zones. The lung tissue was divided to three zones: up (above the carina), middle (between the carina to the inferior pulmonary vein) and down (below the inferior pulmonary vein) with a quantitative score assigned to each zone based on the percentage to be involved： score 0, 0% involvement; score 1, less than 25% involvement; score 2, 25% to less than 50% involvement; score 3, 50% to less than 75% involvement; and score 4, 75% or greater involvement. There were 6 lung zones per patient and therefore the maximal score for each patient was 24.

Lung infection. Ground glass opacity in the lung was defined as hazy areas with slightly increased density in the lung without obscuration of bronchial and vascular margins. Consolidation shadow in the lung was defined when alveolar air was replaced by pathological fluids, cells, or tissues, manifested by an increase in pulmonary parenchymal density that obscures the margins of underlying vessels and airway walls.

RT-PCR. The presence of SARS-CoV-2 was confirmed by real-time RT-PCR. Primers and probes were designed to target the ORF1ab and N genes. Reaction system and amplification conditions were set up according to the manufacturer's specifications (BioGerm, China). Ct-values less than 37 were considered as positive, 40 or more were negative, and those between 37 to 40 were applied to retest. The diagnostic criteria were based on the recommendation by the National Institute for Viral Disease Control and Prevention of China.

Statistical Analysis. All data was presented as mean ± SEM. Comparisons between more than two groups were made using the Kruskal-Wallis's test. Comparisons between two different groups used Mann-Whitney U test. $p \leq 0.05$ (two sided) was considered statistically significant.

Legends for supplemental data

Figure S1: A flow chart for this research work.

Figure S2: Correlation analysis of the Ct Values with concentration of lymphocytes (a, b) or white blood cells (WBC, c, d) in the blood samples of patients at the early stage and the middle stage. NS: non-significant.

Table S1： Clinical characteristics of 77 patients with COVID-19).

Table S2： Comparison of positive detection rates between NS and TS group. NS: asopharyngeal swabs; TS: throat swabs

Supplemental Figure S1

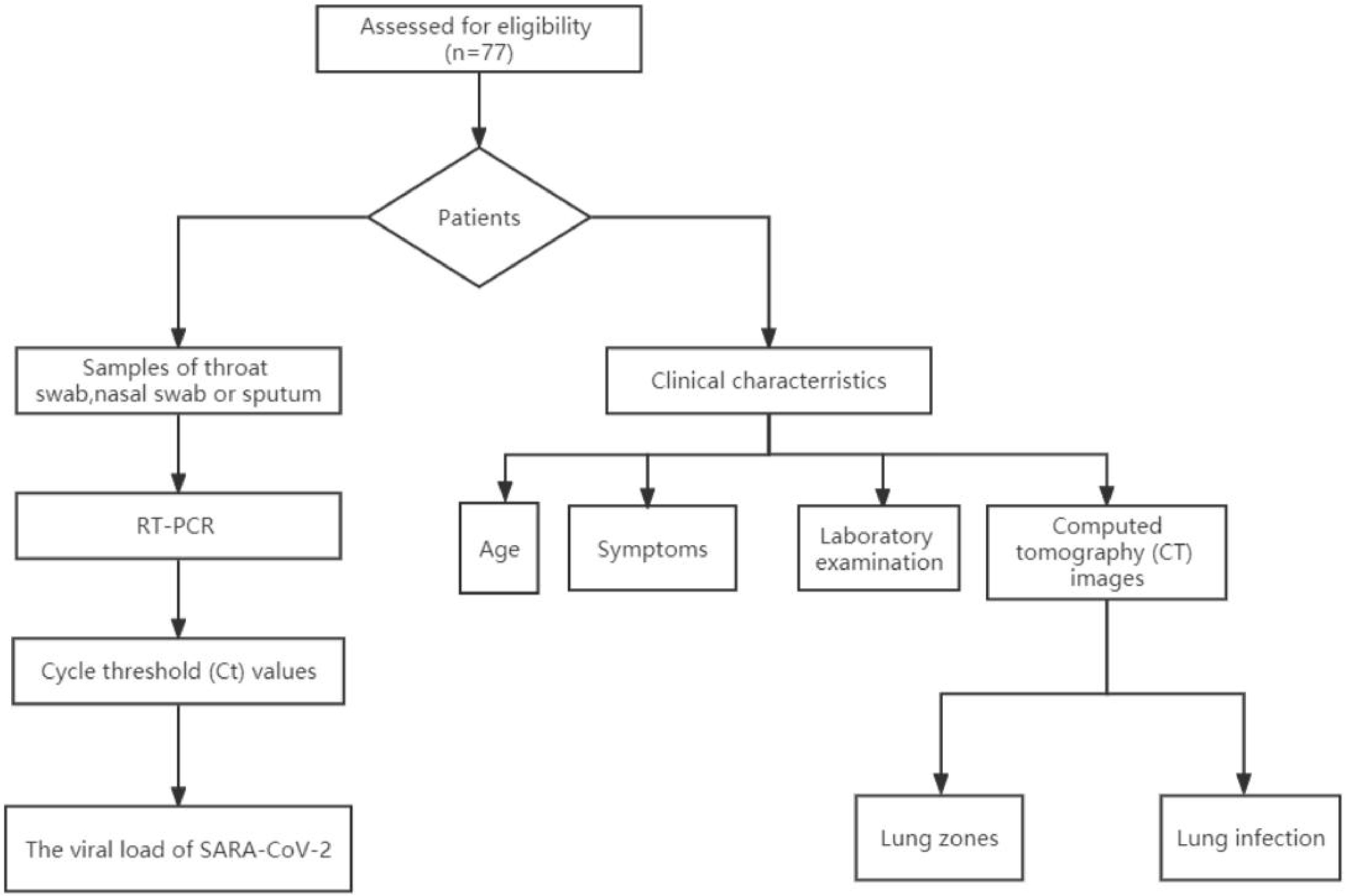

Supplemental Figure S2

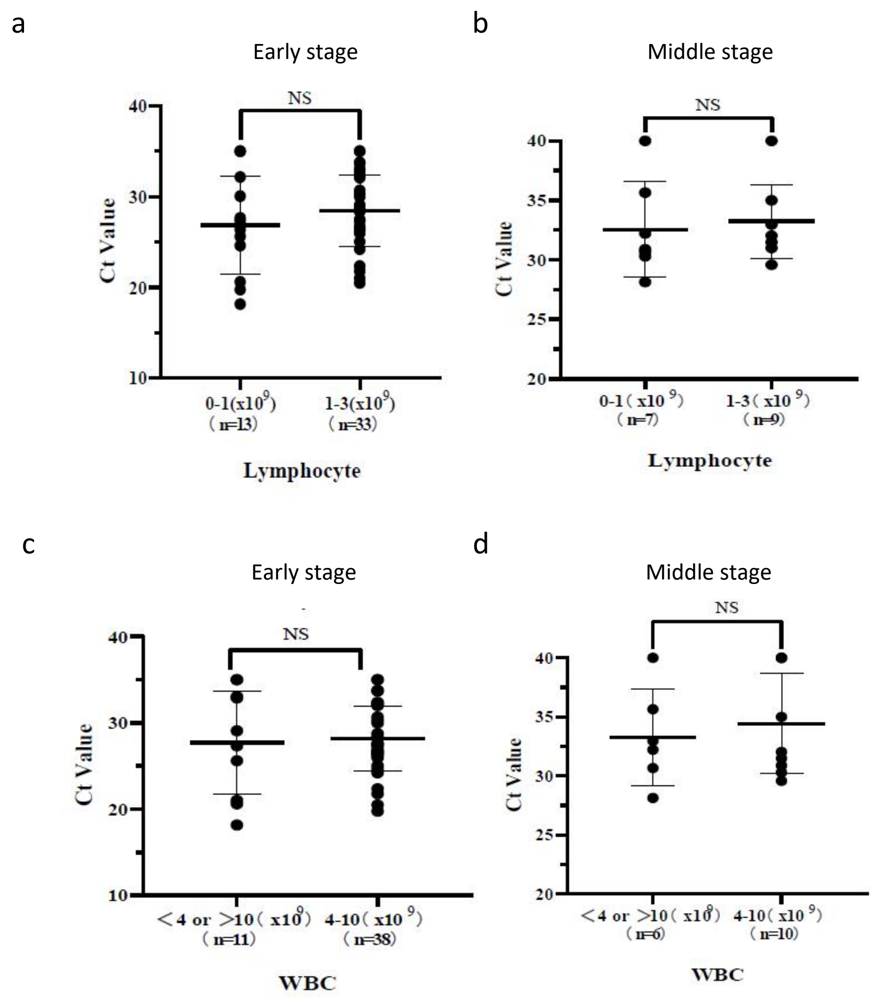

Supplemental Table S1

Table S1： Clinical characteristics of 77 patients with COVID-19 (n = 77)

clinical characteristics	Number (N=77)	Constituent ratio（%）
Gender		
Female	37	48.1
Male	40	51.9
Age（years）		
≤50	34	44.2
＞50	43	55.8
Clinical symptoms		
Fever（≥37.3℃）	66	85.7
Cough	38	49.4
Diarrhea	7	9.1
Muscle weakness	28	36.4
CT score		
0	4	5.2
1～4	46	59.7
5～9	25	32.5
10～12	2	2.6
white blood cells(WBC)（$\times 10^9$/L）		
～10	57	74.0
or ＞10	20	26.0
Lymphocyte counts（$\times 10^9$/L）		
Lymphopenia	8	10.4
Lymphocythemia	10	13.0
Normal	59	76.6
Constitutional symptom		
Fatigue	28	36.4
Sore muscle	12	15.6
Main Imaging		
Ground-glass opacity(GGO)	48	63.3
Lumpy shadow	7	9.1
Patchy shadow	2	2.6
Consolidation shadow	16	20.8
Normal	4	5.2

Supplemental Table S2

Table S2: Comparison of positive detection rates between NS and TS group

Group	n	Positive	Negative	Positive rate (%)
NS	77	66	11	85.7
TS	77	51	26	66.2

NS: nasopharyngeal swabs
TS: throat swabs
NS *vs*TS $p < 0.01$.
